# The IDEAL study: investigation of dietary advice and lifestyle for women with borderline gestational diabetes: a randomised controlled trial - study protocol

**DOI:** 10.1186/1471-2393-12-106

**Published:** 2012-10-09

**Authors:** Caroline A Crowther, William M Hague, Philippa F Middleton, Peter A Baghurst, Andrew J McPhee, Thach S Tran, Lisa N Yelland, Pat Ashwood, Shan Han, Jodie M Dodd, Jeffrey S Robinson

**Affiliations:** 1 Australian Research Centre for Health of Women and Babies (ARCH), The Robinson Institute, Discipline of Obstetrics and Gynaecology, The University of Adelaide, Adelaide, Australia; 2 Discipline of Public Health, The University of Adelaide, Adelaide, Australia; 3 Department of Neonatology, The Women’s and Children’s Hospital, Adelaide, South Australia, Australia; 4 The University of Adelaide, Women’s & Children’s Hospital, 72 King William Road, North Adelaide, 5006, South Australia, Australia

**Keywords:** Borderline gestational diabetes, Gestational diabetes mellitus, Randomised controlled trial, Diet, Lifestyle, Large for gestational age

## Abstract

**Background:**

The Australian Carbohydrate Intolerance Study in Pregnant Women (ACHOIS) showed that treatment of pregnant women with mild gestational diabetes mellitus is beneficial for both women and their infants. It is still uncertain whether there are benefits of similar treatment for women with borderline gestational diabetes.

This trial aims to assess whether dietary and lifestyle advice and treatment given to pregnant women who screen for borderline gestational diabetes reduces neonatal complications and maternal morbidities.

**Methods/design:**

*Design*: Multicentre, randomised controlled trial.

*Inclusion criteria*: Women between 24^0^ and 34^6^ weeks gestation with a singleton pregnancy, a positive oral glucose challenge test (venous plasma glucose ≥7.8 mmol/L) and a normal oral 75 gram glucose tolerance test (fasting venous plasma glucose <5.5 mmol/L and a 2 hour glucose <7.8 mmol/L) with written, informed consent.

*Trial entry and randomisation*: Women with an abnormal oral glucose tolerance test (fasting venous plasma glucose ≥5.5 mmol/L or 2 hour glucose ≥7.8 mmol/L) will not be eligible and will be offered treatment for gestational diabetes, consistent with recommendations based on results of the ACHOIS trial. Eligible women will be randomised into either the ‘Routine Care Group’ or the ‘Intervention Group’.

*Study groups*: Women in the ‘Routine Care Group’ will receive routine obstetric care reflecting current clinical practice in Australian hospitals. Women in the ‘Intervention Group’ will receive obstetric care, which will include dietary and lifestyle advice, monitoring of blood glucose and further medical treatment for hyperglycaemia as appropriate.

*Primary study outcome*: Incidence of large for gestational age infants.

*Sample size*: A sample size of 682 women will be sufficient to show a 50% reduction in the risk of large for gestational age infants (alpha 0.05 two-tailed, 80% power, 4% loss to follow up) from 14% to 7% with dietary and lifestyle advice and treatment.

**Discussion:**

A conclusive trial outcome will provide reliable evidence of relevance for the care of women with borderline glucose intolerance in pregnancy and their infants.

**Trial registration:**

Australian New Zealand Clinical Trials Registry - ACTRN12607000174482

## Background

### Gestational diabetes mellitus: the burden of disease

The prevalence of gestational diabetes mellitus (GDM) is rising worldwide [1,2], with recent Australian estimates indicating that between 5.2% and 8.8% of pregnant women develop GDM [3]. Risk factors for GDM include the mother’s own birthweight, maternal obesity, advanced maternal age, ethnicity, family history of diabetes and a previous history of GDM, large babies or unexplained stillbirth [4-6].

In Australia each year, between 6.3% and 7.7%, or between 15,900 and 19,450 pregnant women have a positive oral glucose challenge test (OGCT) on screening for GDM followed by a normal diagnostic oral glucose tolerance test result (OGTT) [7-9]. These women have borderline gestational diabetes with values of glucose tolerance intermediate between normal and those diagnostic of GDM.

There are well-documented risks for the infant of a mother with GDM including fetal macrosomia, birth injuries such as shoulder dystocia [10], bone fractures and nerve palsies [11], neonatal hypoglycaemia [12], and hyperbilirubinaemia [13]. A later complication associated with macrosomia and large-for-gestational age, for female offspring, is premenopausal breast cancer [14]. Long-term adverse health outcomes reported for the infants include obesity [6,15], impaired intellectual achievement [16], impairment of glucose tolerance [17] and increased risk for subsequent diabetes [6].

For women with GDM there is an increased risk of developing pre-eclampsia and an increased need for induction of labour [18,19]. Impaired glucose tolerance in pregnancy is highly predictive for the later development of diabetes, with over 50% of women with GDM developing type 2 diabetes within 10 years of the index pregnancy [20]. Although the perinatal risks of GDM are well documented, the impact of borderline gestational diabetes on maternal and infant health outcomes is less clear.

We reported a 10-year audit (1993-2003) on a large cohort of women (16,975) at the Women’s and Children’s Hospital, Adelaide, that examined the influence of differing levels of glucose tolerance on pregnancy complications [8]. Women were offered screening for GDM using an oral glucose challenge test (OGCT). Women who screened positive (plasma glucose concentration ≥7.8 mmol/L) were offered a 75 gram diagnostic oral glucose tolerance test (OGTT). Of the women with a positive OGCT who had an OGTT performed, 1074 (6.3% of all women screened) had normal OGTT results. Women with borderline gestational diabetes (positive OGCT, normal OGTT) had a statistically significant increased risk of pre-eclampsia and caesarean section compared with women with normal glucose tolerance (negative OGCT), while the infants of women with borderline gestational diabetes were at increased risk of hypoglycaemia and hyperbilrubinaemia, compared with the infants of women with normal glucose tolerance [8].

The results of our audit are consistent with other observational studies in the literature, identifying an increased risk of adverse maternal and infant outcomes with increasing plasma glucose values [21-23]. A retrospective US study of 1813 women reported an association between increasing levels of hyperglycaemia and the risk of pre-eclampsia. Optimisation of glucose control was found to decrease the risk of pre-eclampsia [23]. The Toronto Tri-Hospital Study screened over 4,000 women, using an OGCT, and if positive, an OGTT, between 26 and 28 weeks gestation, and found increasing degrees of carbohydrate intolerance to be associated with increased risks of pre-eclampsia, caesarean section, macrosomia and need for neonatal phototherapy [21,22]. The international observational study, HAPO, reported an association of increasing hyperglycaemia with greater risks of adverse perinatal outcomes in 25,000 women recruited from over 25 different sites around the world [24].

We reported on the OGCT results and pregnancy outcomes of 1814 women recruited into the ACTS antioxidant supplementation randomised trial (ACTS Trial) [7]. These women were in their first pregnancy and recruited within Australia between 2001 and 2005. The proportion of women with borderline gestational diabetes (positive OGCT, normal OGTT) was 7.7% of all women screened. Women with borderline gestational diabetes (positive OGCT, normal OGTT) had a statistically significant increased risk of serious adverse health outcomes, pregnancy induced hypertension and caesarean section compared with women with normal glucose tolerance (negative OGCT). Infants born to women with borderline gestational diabetes were at increased risk of macrosomia (birthweight ≥4.5 kg) and need for admission to the neonatal nursery.

We consider that there is now compelling evidence of substantially increased risks of adverse health outcomes for both mother and infant when women have borderline gestational diabetes. The question of whether dietary and lifestyle advice and treatment can reduce these risks now requires urgent consideration.

### Treatment of gestational diabetes mellitus improves maternal and infant health

The ACHOIS (Australian Carbohydrate Intolerance Study in Pregnant Women) [18] randomised trial provided evidence that treatment with dietary advice, blood glucose testing and, if required, insulin given to women with mild GDM (fasting glucose <7.0 mmol/L and/or 2-hour glucose ≥7.8 mmol/L but <11.1 mmol/L) significantly reduced the rate of serious perinatal complications (death, shoulder dystocia, bone fracture and nerve palsy) for the infants from 4% to 1%, relative risk (RR), adjusted for maternal age, race and parity, 0.33; 95% confidence interval (CI) 0.14 to 0.75; p=0.01). The number of women needed to treat to prevent one additional serious outcome in an infant was 34 (95% CI, 20 to 103). More infants in the treatment group were admitted to the nursery but there was a significant reduction in the number of infants with a birthweight over 4 kg (10% vs 21%). Women in the treatment group had a higher rate of induction of labour (39% vs 29%) although rates of caesarean section were similar between treatment groups (31% vs 32%). Three months after pregnancy, treated women had significantly lower rates of depression and better scores for health related quality of life [18].

The ACHOIS trial confirmed that mild GDM is a pathological entity that, untreated, is associated with relatively rare but nonetheless significant adverse perinatal outcomes, which can be avoided or reduced with treatment consisting of individualised dietary and lifestyle advice, with insulin treatment as necessary [18]. Cost consequences were in the range acceptable to healthcare funders [25]. The ACHOIS trial provided the first substantial evidence supporting detection and treatment of mild gestational diabetes [26] and has led to proposals for routine screening to become the standard for detection of gestational diabetes in Australia and elsewhere [27-30].

While the ACHOIS trial clarified that treatment of women with mild GDM is beneficial, uncertainty remains whether treatment of women with more borderline gestational diabetes offers similar benefits. Current clinical practice for the management of pregnant women, who screen positive on OGCT but whose subsequent OGTT is normal, is to leave such women ‘untreated,’ with reassurance that their results have not reached the required cut-off for the diagnosis of mild or more severe GDM.

### Critical appraisal of the literature: a Cochrane review

We conducted a systematic review, using the best evidence currently available to assess whether treatment of pregnant women with borderline levels of glucose intolerance (defined as positive OGCT, normal OGTT) improves maternal and infant outcomes [31]. Published randomised controlled trials and cluster-randomised trials comparing alternative management strategies for women with borderline GDM were eligible for inclusion. Pregnant women with hyperglycaemia who did not meet diagnostic criteria for GDM, based on OGTT test results as defined variously by individual trialists according to local health authorities and professional organizations, were considered eligible. Interventions included dietary advice (standard or individualized), exercise and lifestyle advice (standard or individualized) and drug treatment including insulin and oral drugs. Outcomes included both maternal and fetal/neonatal health outcomes, as well as outcomes extending into childhood and adulthood, and health service costs. We used the search strategy of the Cochrane Controlled Trials Register (CENTRAL), MEDLINE, EMBASE, hand-searches of 30 journals and proceedings of major conferences as well as weekly current awareness alerts for a further 44 journals (Date of last search 30 September 2011).

Four studies involving 543 women and their babies were included [32-35]. Two of the four studies were from the United States [33,35], one was from Canada [34] and one from Italy [32]. One study [34] was found to have a low to moderate risk of bias, with the remaining three studies at moderate to high risk of bias. The babies of women receiving management for borderline gestational diabetes were less likely to be macrosomic (birthweight >4000g) (three trials, 438 infants, RR 0.38, 95% CI 0.19 to 0.74) or large-for-gestational age (three trials, 438 infants, RR 0.37, 95% CI 0.20 to 0.66) when compared with those in the routine care group. No significant differences in rates of caesarean section (three trials, 509 women, RR 0.93, 95% CI 0.68 to 1.27) or operative vaginal birth (one trial, 83 women, RR 1.37, 95% CI 0.20 to 9.27) were found.

This review found that for pregnant women with hyperglycaemia who did not meet the diagnostic criteria to be classified as having GDM or type 2 diabetes, interventions such as dietary counselling, blood glucose monitoring and insulin therapy resulted in a reduction in the rates of macrosomia and large for gestational age babies.

Although the results of this systematic review suggest benefit in treating women with borderline gestational diabetes, the review concluded that larger trials are needed of sufficient power and in other populations to assess the effects of management of such women on maternal and infant health outcomes [31].

### Aims and objectives of this trial

It is now clear from the ACHOIS trial [18] that treatment of pregnant women with mild GDM, formerly defined as impaired glucose tolerance, is beneficial for women and their infants. It is still uncertain whether the benefits of similar treatment for women with borderline gestational diabetes outweigh any harms from such treatment.

The aims of this multicentre randomised clinical trial are to assess whether dietary and lifestyle advice and treatment given to pregnant women who have borderline gestational diabetes on screening for GDM (defined as a positive OGCT followed by a normal OGTT), reduces neonatal complications and maternal risks.

### Hypotheses

The primary hypothesis is that dietary and lifestyle advice and treatment given to women who have borderline gestational diabetes on screening for GDM will reduce the incidence of large for gestational age infants, defined as birthweight above the 90^th^ centile for gestation and fetal sex on standardised birthweight charts.

The secondary hypotheses are that dietary and lifestyle advice and treatment given to women who have borderline gestational diabetes will reduce the risk of death or serious health outcome for the infant; reduce the risk of serious health outcome for the woman; reduce the risk of other causes of infant morbidity; and reduce the risks of other adverse health outcomes for the woman.

## Methods/design

### Ethics statement

Ethics approval was granted by the Children’s Youth and Women’s Health Services Human Research Ethics Committee at the Women’s and Children’s Hospital (REC1860/8/09) and by the local institutional review boards for each centre.

### Study design

Multicentre, randomised, controlled trial.

### Inclusion criteria

Women between 24^0^ and 34^6^ weeks gestation with a singleton pregnancy, with a positive OGCT (venous plasma glucose ≥7.8 mmol/L) and a normal 75 gram OGTT (fasting venous plasma glucose <5.5 mmol/L and a 2 hour glucose <7.8 mmol/L), who give written, informed consent.

### Exclusion criteria

Women with known diabetes mellitus, previously treated GDM, active chronic systemic disease (except essential hypertension and mild forms of asthma) or a multiple pregnancy.

### Trial entry

Women who are positive for the OGCT screening will be given the study information sheet, counselled prior to their OGTT, and entered into the trial if they give consent and have a normal OGTT result. Women with an abnormal OGTT result (OGTT fasting ≥5.5 mmol/L or 2-hour ≥7.8 mmol/L) are not eligible for the trial and will be offered treatment for GDM, consistent with recommendations based on the results of the ACHOIS trial [18].

### Study groups and management

Eligible women with borderline gestational diabetes will be randomised into one of two study groups: either the ‘Routine Care Group’ or the ‘Intervention Group’.

### Randomisation

A telephone randomisation service will use a randomisation schedule with balanced variable blocks, prepared by an investigator not involved with recruitment or clinical care. Stratification will be by OGCT result (venous plasma glucose <8.0 mmol/L and ≥8.0 mmol/L) and by collaborating hospital. During the randomisation call, eligibility will be checked and information collected to enable stratification and to assist in follow-up. Information will be collected on baseline demographic characteristics and previous pregnancy outcomes.

### Trial entry questionnaires

At trial entry, women will be asked to complete a questionnaire relating to quality of life (as measured using the SF36 Health Survey Questionnaire) [36], satisfaction with care, anxiety (as measured by the Short Form Spielberger State Trait Inventory) [37], depression (as measured by the Edinburgh Postnatal Depression Scale) [38], physical activity [39] and other lifestyle factors that influence maternal weight gain in pregnancy, including a food frequency questionnaire [40].

### Treatment schedules

#### Intervention group

Women in the ‘Intervention Group’ will be advised that their OGTT results are normal but that they have borderline glucose intolerance. They will receive obstetric care by the attending obstetric team, which will include dietary and lifestyle advice, monitoring of blood glucose and further treatment if appropriate. The intervention is designed to be able to be incorporated into routine antenatal care with minimal additional workload for staff or lengthening of consultation time and to be acceptable to women. Health professionals at the collaborating hospitals have agreed to follow the consensus recommendations below, based on dietary regulation, lifestyle modification and blood glucose monitoring.

#### a) Dietary and lifestyle advice

Women will be given individualised advice regarding their diet from a qualified dietician, based on published recommendations of the Dietitians Association of Australia, which are culturally appropriate and which meet the nutritional requirements of pregnancy. The following characteristics of the woman: age, pre-pregnancy weight, activity level, current dietary intake and weight gain for the current and any previous pregnancies, will be considered in developing an individual woman’s diet and exercise plan. Moderate exercise is recognised as an adjunct to dietary advice [41,42]. Written information will be given to the woman detailing her dietary and exercise goals during pregnancy and will be included in her antenatal study booklet.

#### b) Blood glucose assessments

After trial entry women will have blood glucose monitoring at each antenatal visit consisting of a single, capillary blood glucose (aiming for either fasting or 1 or 2 hours postprandial). Recommended normal blood glucose ranges for women are fasting <5.5 mmol/L, 1 hour postprandial <8.0 mmol/L and 2 hours postprandial <7.0 mmol/L [42]. Indications for obstetric and/or physician review regarding further blood glucose monitoring and treatment are one capillary blood glucose fasting ≥5.5 mmol/L; one capillary blood glucose ≥9.0 mmol/L; or two or more 1 hour postprandial ≥8.0 mmol/L, and/or 2 hour postprandial ≥7.0 mmol/L.

#### c) Further antenatal care

Women will be seen for routine antenatal visits according to standard practice for each hospital. At each visit, progress with their dietary and exercise goals will be reviewed with their health professional and will be recorded in their antenatal study booklet. Care of women will otherwise follow routine clinical practice.

### Routine care group

Women in the ‘Routine Care Group’ will be advised that their OGTT results are normal. They will receive routine obstetric care by the attending obstetric team. Care of women in the ‘Routine Care Group’ will reflect current clinical practice in hospitals in Australia for women who screen positive on OGCT but have normal OGTT results.

### Both study groups

All women in the trial will be asked to complete a questionnaire on health related quality of life [38] at 36 weeks gestation. They will also be asked about their physical activity [39] and dietary habits [40].

### Data collection

Pregnancy, birth and neonatal data will be abstracted from case notes by the research assistant. A standardised checklist determining presence and severity of any shoulder dystocia will be attached to the delivery chart of all women in the study at the time of randomisation, and will be completed by the primary care giver present at the birth.

### Primary study outcomes

The primary study outcome will be incidence of large for gestational age infants defined as birthweight above the 90^th^ centile for gestation and fetal sex on standardised birthweight charts [43].

### Secondary study outcomes

For the infant/child, the secondary study outcomes will be

• death or serious health outcome including one or more of fetal death after trial entry; death of a liveborn infant prior to hospital discharge; severe intrauterine growth restriction (birthweight < 3^rd^ centile for gestation and fetal sex on standardised birth weight charts [43]); severe respiratory distress syndrome (defined as MAP >10 cmH_2_O and or FiO_2_ ≥0.80%); chronic lung disease (defined as need for oxygen at 36 weeks postmenstrual age); intraventricular haemorrhage grade 3 or 4; cystic periventricular leukomalacia; retinopathy of prematurity grade 3 or 4; necrotising enterocolitis; Apgar score <4 at 5 minutes; seizures at <24 hours age or requiring two or more drugs to control; tube feeding ≥4 days; care in neonatal intensive care unit >4 days; use of ventilation for ≥24 hours. These definitions of serious infant outcomes are based on the definitions for adverse outcomes used by the Australian New Zealand Neonatal Network [44] and from those considered by experts as important measures of morbidity at or beyond term [45].

• Other causes of infant morbidity as defined by: individual components of the composite infant outcome; neonatal jaundice requiring phototherapy; neonatal hypoglycaemia requiring treatment (defined as blood glucose <2.5 mmol/L); shoulder dystocia; nerve palsy; bone fracture; gestational age at birth; preterm birth (less than 37 weeks gestation); Apgar score <7 at five minutes; weight, length, head circumference, small for gestational age (<10^th^ percentile for gestation and fetal sex on standardised birthweight charts [43]); macrosomia (≥4 kg); need for admission to the neonatal nursery and length of stay; and need for admission to neonatal intensive care and length of stay; incidence and severity of respiratory distress syndrome, use of and length of mechanical ventilation, intraventricular haemorrhage on early cranial ultrasound, periventricular leukomalacia on later cranial ultrasound, need for oxygen therapy at 28 days or more of life, use of postnatal steroids, use of antibiotics in first 48 hours of life, proven systemic infection in first 48 hours of life, use of antibiotics after first 48 hours of life, proven systemic infection after first 48 hours of life; use of surfactant; nitric oxide for respiratory support; need for inotropic support; air leak syndrome; retinopathy of prematurity; patent ductus arteriosus requiring treatment; number of episodes of proven infection; proven necrotising enterocolitis; thrombocytopenia; neonatal encephalopathy (Sarnat Stage 1, 2 or 3) [46].

For the mother, the secondary outcomes will be

• serious health outcomes up to six weeks postpartum as defined by one or more of maternal death; pulmonary oedema; eclampsia; stroke; adult respiratory distress syndrome; cardiac arrest; respiratory arrest; placental abruption; haemolysis; coagulopathy; major postpartum haemorrhage; deep vein thrombosis or pulmonary embolism requiring anticoagulant therapy.

• other adverse health outcomes as defined by: individual components of the composite maternal outcome; pre-eclampsia [47]; caesarean birth; induction of labour; need for antenatal hospitalization and length of stay; antepartum haemorrhage requiring hospital admission; weight gain during pregnancy; use of antihypertensive medication; chorioamnionitis requiring antibiotics during labour; length of postnatal hospital stay; use of postnatal antibiotics; and postpartum haemorrhage (≥500mls).

• Maternal diet and exercise outcomes as measured by dietary and exercise [39] questionnaires at trial entry and 36 weeks gestation.

### Sample size

The incidence of having a large for gestational age infant is the principal endpoint of the trial. In the Italian trial [32], 14% of infants born to women in the untreated group were large for gestational age compared with 6% in the infants of treated women, a 57% change. A trial of 682 women will be able to show a more conservative 50% reduction in risk of large for gestational age infants from 14% to 7% with dietary and lifestyle advice and treatment (5% level of significance, two – tailed alpha, 80% power, 4% loss to follow up).

This size of trial will be powered also to detect important differences in key secondary outcomes. The rate of serious health outcome for both the infant and the woman is 12.9% based on data from women with untreated borderline GDM in the Australasian Collaborative Trial of Supplements with vitamin C and vitamin E for the prevention of pre-eclampsia (ACTS) [7]. The sample size of 682 women will allow a 51% reduction in the risk of serious maternal and infant health outcome from 12.9% to 6.3% to be detected with 80% power.

### Analyses and reporting of results

An independent data monitoring committee will be established, with terms of reference. A multidisciplinary adverse events committee blinded to treatment allocation will review the cause of death for all maternal and infant deaths. These data will be made available to the independent Data Monitoring Committee. Recruitment will continue until sample size is reached followed by completion of data collection and analyses.

The analyses will follow several key steps. Baseline characteristics of all randomised women will be compared descriptively between the study groups. Outcome comparisons will be made according to the treatment allocation at randomisation on an ‘intention to treat’ basis. Both unadjusted and adjusted analyses will be carried out. Collaborating centre and OGCT result will be controlled for in all adjusted analyses. The primary outcome of large for gestational age and the secondary outcomes based on birth weight will be adjusted for maternal age, parity, body mass index, socioeconomic status and gestational age at entry.

Secondary exploratory analyses will consider baseline covariates that show evidence of imbalance between study groups and are related to the outcome of interest. The RRs and 95% CIs will be reported using log binomial regression for binary outcomes. The number needed to treat to benefit or harm for one adverse outcome will be calculated for the primary outcome. Continuous outcomes will be analysed using linear regression. All model assumptions, including normality, will be assessed. Statistical significance will be assessed at the 0.05 level using a two-sided comparative test.

## Discussion

This project is a multicentre, randomised clinical trial assessing whether dietary and lifestyle advice and treatment given to women with borderline gestational diabetes reduces perinatal morbidity, and evaluates the effects of such treatment on maternal physical and psychological morbidity.

Systematic review of the literature suggests a reduction in the risk of large for gestational age infants born to women receiving treatment for borderline gestational diabetes. There have, however, been only four small trials reported to date. Larger, high quality trials of sufficient power to detect differences in clinically relevant maternal and infant health outcomes are a priority.

This trial will provide evidence on which to guide practice for the care of women and their infants in these circumstances. This trial is of considerable importance given more than 16,000 women are affected each year in Australia alone. There is continuing uncertainty about diagnosis and treatment of borderline gestational diabetes and a lack of randomised controlled trials to date.

Until data from large, well-designed randomised trials are available to assess this intervention, it is difficult to develop meaningful, evidence-based, clinical practice guidelines. A conclusive outcome will provide important, reliable evidence of great relevance for the care of the significant number of women with borderline gestational diabetes in pregnancy and their infants.

## Abbreviations

ACHOIS: Australian Carbohydrate Intolerance Study in Pregnant Women; ACTS: Australasian Collaborative Trial of Supplements with vitamin C and vitamin E for the prevention of pre-eclampsia; CI: Confidence interval; GDM: Gestational diabetes mellitus; OGCT: Oral glucose challenge test; OGTT: Oral glucose tolerance test; RR: Relative risk.

## Competing interests

The authors declare they have no competing interests.

## Authors' contributions

CAC, WMH, PFM, PAB, AJMc, TST, LNY, JMD, JSR, PA and SH are all members of the IDEAL Study Group. The primary investigator of the IDEAL Study (CAC) prepared the initial draft of the IDEAL protocol. The investigators (CAC, WMH, PFM, PAB, AJMc, JMD, JSR) participated in the design of the study. The IDEAL Study Group participated in the protocol development, commented on drafts of the protocol, and have read and approved the final draft of the protocol. All authors read and approved the final manuscript.

## Pre-publication history

The pre-publication history for this paper can be accessed here:

http://www.biomedcentral.com/1471-2393/12/106/prepub
